# Supplementary material for: Fitness Costs and Incomplete Resistance Associated with Delayed Evolution of Practical Resistance to Bt Crops
Source: Insects. 2023 Feb 21;14(3):214. doi: 10.3390/insects14030214 (PMC10051223; doi:10.3390/insects14030214)
Supplement: Supplementary file 1 [file insects-14-00214-s001.zip › File S1 and Table S3.pdf]

Fitness costs and incomplete resistance associated with delayed evolution of practical resistance to Bt crops

Yves Carrière and Bruce E. Tabashnik

Supplementary material

File S1: Statistical Analyses

Fitness Costs

We used multiple regression to compare the relative fitness of the resistant strain or F_1 progeny relative to the susceptible strain (i.e., $W_{R/S}$ or $W_{RS/S}$) between cases of pests with and without practical resistance. The response variable was $W_{R/S}$ (square root [X + 0.5] transformed) or $W_{RS/S}$ (log transformed). We used different transformations of these response variables based on their capacity to improve assumptions of normality and homogeneity of variance. Explanatory variables in the basic model were strain relatedness (susceptible and resistant strains related or not), food type (artificial diet, corn or cotton), and species and country (e.g., *Helicoverpa armigera* from Australia) nested within food type. Strains were considered related if the resistant strain was selected from the susceptible strain (n = 34 cases, 35% of cases), ≥ 4 backcrosses followed by selection were used to homogenize the genetic background of the susceptible and resistant strains (n = 12 cases, 12% of cases), or costs were estimated from genotypes in hybrid strains identified with PCR (n = 7 cases, 7% of cases).

We tested for potential influence of two additional factors on $W_{R/S}$ or $W_{RS/S}$: the magnitude of resistance to the Bt toxin (estimated by the resistance ratio [RR] obtained from diet bioassays, log transformed) and the type of traits used to estimate costs (Ro or fitness components). When entered one at a time in the basic model, neither of these variables were significantly associate with $W_{R/S}$ (log RR: slope = -0.009, df = 1, 38, t = -0.27, P = 0.79; type of fitness measures: df = 1, 75, F = 0.070, P = 0.79) or $W_{RS/S}$ (log RR: slope = 0.0016, df = 1, 23, t = 0.13, P = 0.90; type of fitness measures: df = 1, 46, F = 2.40, P = 0.13). Thus, these explanatory variables were not included in analyses to compare $W_{R/S}$ or $W_{RS/S}$ between cases with and without practical resistance.

Least squares means for species from particular countries obtained from the basic models are adjusted statistically for effects of strain relatedness and food type. We used contrasts between these least squares means to test the hypotheses that $W_{R/S}$ or $W_{RS/S}$ differed significantly between cases with and without practical resistance. Back-transformed least squares means were used to estimate the average costs and associated 95% confidence interval for cases with and without practical resistance.

Incomplete Resistance

We used multiple regression to evaluate whether IR (square root [X + 0.5] transformed) differed significantly between cases with and without practical resistance. Explanatory variables included in the basic model were food type (corn or cotton) and species and country nested within food type. The magnitude of costs could affect IR because costs affect survival of resistant strains on the non-Bt crop. We thus assessed whether the magnitude of costs (i.e., $W_{R/S}$) or the extent of resistance (i.e., the resistance ratio, log transformed) were associated with IR. When entered one at a time in the basic model, these variables were not significantly associate with IR (log RR: slope = - 0.0082, df = 1, 9, F = 0.83, P = 0.84; $W_{R/S}$: df = 1, 21, F = 1.33, P = 0.26). These variables were not included in the model used to test whether IR differed significantly between cases with and without practical resistance with least squares means contrasts.

Table S1. Estimates of relative fitness of resistant strains or F_1 progeny relative to susceptible strains ($W_{R/S}$ and $W_{RS/S}$) from literature review (attached excel file)

Table S2. Estimates of incomplete resistance (IR) from literature review (attached Excel file)

Table S3. Fitness of the genotypes on the non-Bt and Bt crop used in simulations. Recessive fitness costs on the non-Bt crop were 0, 14 or 30%. Incomplete resistance on the Bt crop was 1 (i.e., 100% survival and no incomplete resistance), 0.76 or 0.43. With non-recessive resistance (*h* = 0.26), fitness of *rs* was adjusted to keep the dominance of resistance constant across levels of incomplete resistance.

**Crop**	**Cost (%)**	**Fitness**		
		Rr	*rs*	*ss*
Non-Bt	0	1	1	1
	14	0.86	1	1
	30	0.70	1	1

**Crop**	**Dominance of resistance (*h*)**	**Incomplete resistance**	**Fitness**		
			rr	*rs*	*ss*
Bt	0	1	1	0.01	0.01
		0.76	0.76	0.01	0.01
		0.43	0.43	0.01	0.01
	0.26	1	1	0.26	0.01
		0.76	0.76	0.21	0.01
		0.43	0.43	0.12	0.01

References for Tables S1 and S2

Acharya, B.; Head, G.P.; Price, P.A.; Huang, F. Fitness costs and inheritance of Cry2Ab2 resistance in *Spodoptera frugiperda* (J.E. Smith). *J. Invertebr. Pathol.* **2017**, *149*, 8–14. http://dx.doi.org/10.1016/j.jip.2017.06.014

Akhurst, R.J.; James, W.; Bird, L.J.; Beard, C. Resistance to the Cry1Ac-Endotoxin of *Bacillus thuringiensis i*n the cotton bollworm, *Helicoverpa armigera* (Lepidoptera: Noctuidae). *J. Econ. Entomol.* **2003**, *96*, 1290-1299. http://doi.org/10.1603/0022-0493-96.4.1290

Bernardi, O.; Bernardi, D.; Horikoshi, R.J.; Okuma, D.M.; Miraldo, L.L.; Fatoretto, J.; Medeiros, F.C.L.; Burd, T.; Omoto, C. Selection and characterization of resistance to the Vip3Aa20 protein from *Bacillus thuringiensis* in *Spodoptera frugiperda. Pest. Manag. Sci.* **2016**, *72,* 1794–1802. http://doi.org/10.1002/ps.4223

Bird, L.J.; Akhurst, R.J. Relative fitness of Cry1A-resistant and –susceptible *Helicoverpa armigera* (Lepidoptera: Noctuidae) on conventional and transgenic Cotton. *J. Econ. Entomol.* **2004**, *97*, 1699-1709. http://doi.org/10.1603/0022-0493-97.5.1699

Bird, L.J.; Akhurst, R.J. Fitness of Cry1A-resistant and -susceptible *Helicoverpa armigera* (Lepidoptera: Noctuidae) on transgenic cotton with reduced levels of Cry1Ac. *J. Econ. Entomol.* **2005**, *98*, 1311-1319. https://doi.org/10.1603/0022-0493-98.4.1311

Brévault, T.; Heuberger, S.; Zhang, M.; Ellers-Kirk, C.; Ni, X.; Masson, L.; Li, X.; Tabashnik, B. E.; Carrière, Y. Potential shortfall of pyramided Bt cotton for resistance management. *Proc. Natl. Acad. Sci. USA.* **2013**, *110*, 5806-5811. https://doi.org/10.1073/pnas.1216719110

Carrière, Y.; Ellers-Kirk, C.; Liu, Y.-B.; Sims, M.A.; Patin, A.L.; Dennehy, T.J.; Tabashnik, B.E. Fitness costs and maternal effects associated with resistance to transgenic cotton in the pink bollworm (Lepidoptera: Gelechiidae). *J. Econ. Entomol.* **2001**, *94*, 1571-1576. https://doi.org/10.1603/0022-0493-94.6.1571

Carrière, Y.; Ellers-Kirk, C.; Biggs, R.; Higginson, D.M.; Dennehy, T.J.; Tabashnik, B.E. Effects of gossypol on fitness costs associated with resistance to Bt cotton in pink bollworm. *J. Econ. Entomol.* **2004**, *97*, 1710-1718. http://doi.org/10.1603/0022-0493-97.5.1710

Carrière, Y.; Ellers-Kirk, C.; Biggs, R.; Degain, B.; Holley, D.; Yafuso, C.; Evans, P.; Dennehy, T.J.; Tabashnik, B.E. Effects of cotton cultivar on fitness costs associated with resistance of pink bollworm (Lepidoptera: Gelechiidae) to Bt cotton. *J. Econ. Entomol.* **2005,** *98*, 947-954. https://doi.org/10.1603/0022-0493-98.3.947

Carrière, Y.; Ellers-Kirk, C.; Biggs, R.; Nyboer, M.E.; Unnithan, G.C.; Dennehy, T.J.; Tabashnik, B.E. Cadherin-based resistance to *Bacillus thuringiensis* cotton in hybrid strains of pink bollworm: Fitness costs and incomplete resistance. *J. Econ. Entomol.* **2006**, *99*, 1925-1935. http://doi.org/10.1603/0022-0493-99.6.1925

Carrière, Y.; Showalter, A.M.; Fabrick, J.A.; Sollome, J.; Ellers-Kirk, C.; Tabashnik, B.E. Cadherin gene expression and effects of Bt resistance on sperm transfer in pink bollworm. *J. Ins. Physiol.* **2009,** *55*, 1058–1064. http://doi.org/10.1016/j.jinsphys.2009.07.013

Carrière, Y.; Degain, B.A.; Unnithan, G.C.; Harpold, V.S.; Heuberger, S.; Li X.; Tabashnik, B.E. Effects of seasonal changes in cotton plants on the evolution of resistance to pyramided cotton producing the Bt toxins Cry1Ac and Cry1F in *Helicoverpa zea*. *Pest. Manag. Sci.* **2018**, *74*, 627–637. http://doi.org/10.1002/ps.4746

Carrière, Y.; Degain, B.; Unnithan, G. C.; Harpold, V.; Heuberger, S.; Li, X.; Tabashnik, B. E. Effects of seasonal changes in cotton plants on evolution of resistance to pyramided cotton producing the toxins Cry1Ac and Cry1F by *Helicoverpa zea*. *Pest Manag. Sci.* **2019**, *74*, 627–637. https://doi.org/10.1002/ps.4746

Carrière, Y.; Degain, B.A.; Tabashnik, B.E. Effects of gene flow between Bt and non-Bt plants in a seed mixture of Cry1A.105 + Cry2Ab corn on performance of corn earworm in Arizona. *Pest. Manag. Sci.* **2021**, *77*, 2106–2113. http://doi.org/10.1002/ps.6239

Chen, X.; Head, G.P.; Price, P.; Kerns, D.L.; Rice, M.E.; Huang, F.; Gilreath, R.T.; Yang, F. Fitness costs of Vip3A resistance in *Spodoptera frugiperda* on different hosts. *Pest Manag. Sci.* **2019,** *75*, 1074–1080. https://doi.org/10.1002/ps.5218

Crespo, A.L.B.; Spenser, T.A.; Tan, S.Y.; Siegfried, B.D. Fitness Costs of Cry1Ab Resistance in a Field-Derived Strain of *Ostrinia nubilalis* (Lepidoptera: Crambidae). *J. Econ. Entomol.* **2010**, *103*, 1386-1393. http://doi.org/10.1603/EC09158

Crickmore, N.; Berry, S.; Panneerselvam, S.; Mishra, R.; Connor, T.R.; Bonning, B.C.

A structure-based nomenclature for *Bacillus thuringiensis* and other bacteria-derived

pesticidal proteins. *J. Invertebr. Pathol.* **2020**, *6*, 107438.
https://doi.org/10.1016/j.jip.2020.107438

Dangal, V.; Huang, F. Fitness costs of Cry1F resistance in two populations of fall armyworm, *Spodoptera frugiperda* (J.E. Smith), collected from Puerto Rico and Florida. *J. Invertebr. Pathol.* **2015**, *127*, 81–86. http://dx.doi.org/10.1016/j.jip.2015.03.004

de Souza Ribas, N.; McNeil, J.N.; Araújo, H.D.; de Souza Ribas, B.; Lima, E. The Effect of resistance to Bt corn on the reproductive output of *Spodoptera frugiperda* (Lepidoptera: Noctuidae). *Insects*. **2022**, *13*, 196. https://doi.org/10.3390/insects13020196

Ghimire, M.N.; Huang, F.; Leonard, R.; Head, G.P.; Yang, Y. Susceptibility of Cry1Ab-susceptible and -resistant sugarcane borer to transgenic corn plants containing single or pyramided Bacillus thuringiensis genes. *Crop Prot.* **2011**, *30*, 74e81.
https://doi.org/10.1016/j.cropro.2010.09.008

Geisert, R.W.; Hibbard, B.E. Evaluation of potential fitness costs associated with eCry3.1Ab resistance in *Diabrotica virgifera virgifera* (Coleoptera: Chrysomelidae). *J. Econ. Entomol.* **2016**, *109*, 1853–1858. http://doi.org/10.1093/jee/tow095

Gulzar, A.; Pickett, B.; Sayyed, A.H.; Wright, D.J. Effect of temperature on the fitness of a Vip3A resistant population of Heliothis virescens (Lepidoptera: Noctuidae). *J. Econ. Entomol.* **2012**, *105*, 964-970. http://dx.doi.org/10.1603/EC11110

Higginson, D.M.; Morin, S.; Nyboer, M.E.; Biggs, R.W.; Tabashnik, B.E.; Carrière, Y. Evolutionary trade-offs of insect resistance to *Bacillus thuringiensis* crops: Fitness cost affecting paternity. *Evolution*. **2005**, *59*, 915–920. https://doi.org/10.1554/04-737

Horikoshi, R.J.; Bernardi, O.; Bernardi, D.; Okuma, D.M.; Farias, J.R.; Miraldo, L.L.; Amaral, F.S.A.; Omoto, C. Near-isogenic Cry1F-resistant strain of *Spodoptera frugiperda* (Lepidoptera: Noctuidae) to investigate fitness cost associated with resistance in Brazil. *J. Econ. Entomol.* **2016**, *109*, 854–859. http://doi.org/10.1093/jee/tov387

Huang, F.; Chen, M.:, Gowda, A.; Clark, T.L.; McNulty, B.C.; Yang, F.; Niu, Y. Identification, inheritance, and fitness costs of Cry2Ab2 resistance in a field-derived population of sugarcane borer, *Diatraea saccharalis* (F.) (Lepidoptera: Crambidae). *J. Invertebr. Pathol.* **2015**, *130*, 116–123. http://dx.doi.org/10.1016/j.jip.2015.07.007

Hoffmann, A.M.; French, B.W.; Hellmich, R.L.; Lauter, N.; Gassmann, A.J. Fitness costs of resistance to Cry3Bb1 maize by western corn rootworm. *J. Appl. Entomol.* **2015**, *139*, 403–415. http://doi.org/10.1111/jen.12209

Ingber, D.A; Gassmann, A.J. Inheritance and fitness costs of resistance to Cry3Bb1 corn by western corn rootworm (Coleoptera: Chrysomelidae). *J. Econ. Entomol*. **2015**, *108*, 2421–2432. http://doi.org/10.1093/jee/tov199

Jackson, R.E.; Bradley, J.R.; Van Duyn, J.W. Performance of feral and Cry1Ac-selected *Helicoverpa zea* (Lepidoptera: Noctuidae) strains on transgenic cottons expressing one or two *Bacillus thuringiensis* ssp. Kurstaki proteins under greenhouse conditions. *J. Entomol. Sci.* **2004**, *39*, 46-55. https://doi.org/10.18474/0749-8004-39.1.46

Jakka, S.R.K.; Knight, V.R.; Jurat-Fuentes, J.L. Fitness costs associated with field-evolved resistance to Bt maize in *Spodoptera frugiperda* (Lepidoptera: Noctuidae). *J. Econ. Entomol.* **2014**, *107,* 342-351. http://dx.doi.org/10.1603/EC13326

Kruger, M.; Van Rensburg, J.B.J; Van den Berg, J. No fitness costs associated with resistance of *Busseola fusca* (Lepidoptera: Noctuidae) to genetically modified Bt maize. *Crop Protect*. **2014,** *55*, 1-6. http://dx.doi.org/10.1016/j.cropro.2013.09.004

Lefko, S.A.; Nowatzki, T.M.; Thompson, S.D.; Binning, R.R.; Pascual, M.A.;Peters, M.L.; Simbro, E.J.; Stanley, B.H. Characterizing laboratory colonies of western corn rootworm (Coleoptera: Chrysomelidae) selected for survival on maize containing event DAS-59122-7. *J. Appl. Entomol.* **2008**, *132*, 189–204. http://doi.org/10.1111/j.1439-0418.2008.01279.x

Leite, N.A.; Mendes, S.M.; Santos-Amaya, O.F.; Santos, C.A.;Teixeira, T.P.M.; Guedes, R.N.C.; Pereira, E.J.G. Rapid selection and characterization of Cry1F resistance in a Brazilian strain of fall armyworm. *Entomol. Exp. Appl*. **2016**, *158*, 236–247. http://doi.org/10.1111/eea.12399

Liang, G.-M.; Wu, K.-M.; Rector, B.; Guo, Y.-Y. Diapause, cold hardiness and flight ability of Cry1Ac-resistant and –susceptible strains of *Helicoverpa armigera* (Lepidoptera: Noctuidae). *Eur. J. Entomol.* **2007,** *104*, 699–704. http://www.eje.cz/scripts/viewabstract.php?abstract=1277

Liang, G.-M.; Wu, K.-M.; Yu, H.K.; Li, K.-K.; Feng, X.; Guo, Y.-Y. Changes of inheritance mode and fitness in *Helicoverpa armigera* (Hubner) (Lepidoptera: Noctuidae) along with its resistance evolution to Cry1Ac toxin. *J. Invertebr. Pathol.* **2008**, *97*, 142–149. http://doi.org/10.1016/j.jip.2007.09.007

Lin, S.; Head, G.; Price, P.; Niu, Y.; Huang, F. Relative fitness of susceptible and Cry1A.105/Cry2Ab2-single-/dual-protein-resistant *Helicoverpa zea* (Boddie)(Lepidoptera: Noctuidae) on non-Bt diet and a diet containing a low concentration of two proteins. *Ins. Sci.* **2022**, 0, 1–13. https://doi.org/10.1111/1744-7917.13087

Liu, Y.-B.; Tabashnik, B.E.; Dennehy, T.J.; Patin, A.L.; Bartlett, A.C. Development time and resistance to Bt crops. *Nature*. **1999**, *400*, 519. https://doi.org/10.1038/22919

Liu, Y.-B.; Tabashnik, B.E.; Dennehy, T.J.; Patin, A.L.; Sims, M.A.; Meyer, S.K.; Carrière, Y. Effects of Bt cotton and Cry1Ac toxin on survival and development of pink bollworm (Lepidoptera: Gelechiidae). *J. Econ. Entomol.* **2001**, *94*, 1237-1242. http://doi.org/10.1603/0022-0493-94.5.1237

Mahon, R.J.; Olsen, K.M. Limited survival of a Cry2Ab-resistant strain of *Helicoverpa armigera* (Lepidoptera: Noctuidae) on Bollgard II. *J. Econ. Entomol.* **2009**, *102*, 708-716. https://doi.org/10.1603/029.102.0232

Malthankar, P.A.; Gujar, G.T. Toxicity of *Bacillus thuringiensis* Cry2Ab and inheritance of Cry2Ab resistance in the pink bollworm, *Pectinophora gossypiella* (Saunders). *Indian. J. Exp. Biol.* **2016**, *54*, 586-596.

Meihls, L.N.; Higdon, M.L.; Ellersieck, M.R.; Tabashnik, B.E.; Hibbard, B.E. Greenhouse-selected resistance to Cry3Bb1-producing corn in three western corn rootworm populations. *PLoS ONE,* **2012**, *7:* e51055. http://doi.org/10.1371/journal.pone.0051055

Meihls, L.N., Frank, D.L., Ellersieck, L.R.; Hibbard, B.E. Development and characterization of MIR604 resistance in a western corn rootworm population (Coleoptera: Chrysomelidae). *Environ. Entomol.* **2016**, *45*, 526–536. http://doi.org/10.1093/ee/nvv226

Mittal, A.: Ningthoukongjan, J.; Malthankar, P.A.; Ningthoujam, K.; Kalia, V.; Gujar. G.T. Toxicity of Cry1Ac, inheritance of Cry1Ac resistance and fitness costs association in the pink bollworm, *Pectinophora gossypiella* (Lepidoptera: Gelechiidae). *Biopestic. Int.* **2016**, *12*, 127-138.

Niu, Y.; Head, G.P.; Price, P.A.; Huang, F. Inheritance and fitness costs of Cry1A.105 resistance in two strains of *Spodoptera frugiperda* (J.E. Smith). *Crop Prot.* **2018**, *110*, 229-235. http://dx.doi.org/10.1016/j.cropro.2017.06.022

Orpet, R.J.; Degain, B.A.; Unnithan, G.C.; Welch, K.L.; Tabashnik, B.E.; Carrière, Y. Effects of dietary protein to carbohydrate ratio on Bt toxicity and fitness costs of resistance in *Helicoverpa zea. Entomol. Exp. Appl.* **2015**, *156,* 28–36. http://doi.org/10.1111/eea.12308

Oswald, K.J; French, B.W.; Nielson, C.; Bagley, M. Assessment of fitness costs in Cry3Bb1-resistant and susceptible western corn rootworm (Coleoptera: Chrysomelidae) laboratory colonies. *J. Appl. Entomol.* **2012**, *136*, 730–740. http://doi.org/10.1111/j.1439-0418.2012.01704.x

Paddock, K.J.; Hibbard, B.E.; Barry, J.; Sethi, A.; Mueller, A.L.; Shelbyd, K.J.; Pereira, A.E. Restoration of susceptibility following removal of selection for Cry34/35Ab1 resistance documents fitness costs in resistant population of western corn rootworm, *Diabrotica virgifera virgifera. Pest. Manag. Sci.* **2021**, *77*, 2385–2394. http://doi.org/10.1002/ps.6266

Paolino, A.R.; Gassmann, A.J. Assessment of inheritance and fitness costs associated with field-evolved resistance to Cry3Bb1 maize by western corn rootworm. *Toxins*, **2017**, *9,* 159. http://doi.org/doi:10.3390/toxins9050159

Pereira, E. J. G.; Storer, N.P.; Siegfried, B.D. Fitness costs of Cry1F resistance in laboratory-selected European corn borer (Lepidoptera: Crambidae). *J. Appl. Entomol.* **2011**, *135*, 17–24. http://doi.org/10.1111/j.1439-0418.2009.01488.x

Petzold-Maxwell, J.L.; Cibils-Stewart,X.; Wade French, B.; Gassmann, A.J. Adaptation by western corn rootworm (Coleoptera: Chrysomelidae) to Bt maize: Inheritance, fitness costs, and feeding preference. *J. Econ. Entomol.* **2012**, *105*, 1407-1418. http://dx.doi.org/10.1603/EC11425

Petzold-Maxwell, J.L.; Siegfried, B.D.; Hellmich, R.L.; Abel, C.A.; Coates, B.S.; Spencer, T.A.; Gassmann, A.J. Effect of maize lines on larval fitness costs of Cry1F resistance in the European corn borer (Lepidoptera: Crambidae*). J. Econ. Entomol.* **2014**, *107*, 764-772. http://dx.doi.org/10.1603/EC13359

Petzold-Maxwell, J.L.; Siegfried, B.D.; Hellmich, R.L.; Abel, C.A.; Coates, B.S.; Spencer, T.A.; Horikoshi, R.J.; Gassmann, A.J. Fitness costs associated with Cry1F resistance in the European corn borer. *J. Appl. Entomol*. **2017**, *141*, 67–79. https://doi.org/10.1111/jen.12356

Santos-Amaya, O.F.; Tavares, C.S.;Monteiro, H.M.; Teixeira, T.P.M.; Guedes, R.N.C.; Alves, A.P.; Pereira, E.J.G. Genetic basis of Cry1F resistance in two Brazilian populations of fall armyworm, *Spodoptera frugiperda*. *Crop Prot.* **2016**, *81*, 154-162. http://dx.doi.org/10.1016/j.cropro.2015.12.014

Santos-Amaya, O.F.; Tavares, C.S.; Rodrigues, J.V.C.; Campos, S.O.; Guedes, R.N.C.; Alves, A.P.; Pereira, E.J.G. Fitness costs and stability of Cry1Fa resistance in Brazilian populations of *Spodoptera frugiperda*. *Pest. Manag. Sci.* **2017**, *73*, 35–43. https://doi.org/10.1002/ps.4312

Shrestha, R.B.; Gassmann, A.J. Inheritance and fitness costs of Cry3Bb1 resistance in diapausing field strains of western corn rootworm (Coleoptera: Chrysomelidae). *J. Econ. Entomol*. **2020**, *113*, 2873–2882. http://doi.org/10.1093/jee/toaa213

van Rensburg, J.B.J. First report of field resistance by the stem borer, *Busseola fusca* (Fuller) to Bt-transgenic maize. *South Afr. J. Plant and Soil.* **2007**, *24*, 147-151. http://doi.org/10.1080/02571862.2007.10634798

Vélez, A.M.; Spencer, T.A.; Alves, A.P.; Crespo, A.L.B.; Siegfried, B.D. Fitness costs of Cry1F resistance in fall armyworm, *Spodoptera frugiperda*. *J. Appl. Entomol*. **2014**, *138*, 315–325. http://doi.org/10.1111/jen.12092

Wade French, B.; Hammack, L.; Tallamy, D.W. Mating success, longevity, and fertility of *Diabrotica virgifera virgifera* LeConte (Chrysomelidae: Coleoptera) in relation to body size and Cry3Bb1-resistant and Cry3Bb1-susceptible genotypes. *Insects*. **2015**, *6*, 943-960. http://doi.org/10.3390/insects6040943

Wangila, D.S.; Leonard, B.R.; Bai, Y.: Head, G.P.; Huang, F. Larval survival and plant injury of Cry1Ab-susceptible, -resistant, and -heterozygous genotypes of the sugarcane borer on transgenic corn containing single or pyramided Bt genes. *Crop Prot.* **2012**, *42*, 108-115. http://dx.doi.org/10.1016/j.cropro.2012.06.005

Williams, J.L.; Ellers-Kirk, C.; Orth, R.G.; Gassmann, A.J.; Head, G.; Tabashnik, B.E.; Carrière, Y. Fitness cost of resistance to Bt cotton linked with increased gossypol content in pink bollworm larvae. *PLoS ONE*. **2010**, *6*, e21863. http://doi.org/10.1371/journal.pone.0021863

Wu, X.; Huang, F.; Leonard, B.R.; Ghimire, M. Growth and development of *Bacillus thuringiensis* Cry1Ab-susceptible and Cry1Ab-resistant sugarcane borer on diet and conventional maize plants. *Entomol. Exp. Appl.* **2009**, *133*, 199–207. http://doi.org/10.1111/j.1570-7458.2009.00919.x

Yang, Y.-H.; Yang, Y.-J.; Gao, W.-Y.; Guo, J.-J.; Wu, Y.-H.; Wu, Y.D. Introgression of a disrupted cadherin gene enables susceptible *Helicoverpa armigera* to obtain resistance to *Bacillus thuringiensis* toxin Cry1Ac. *Bull. Entomol. Res.* **2009**, *99*, 175–181. http://doi.org/10.1017/S0007485308006226

Zhang, L.: Leonard, B.R.; Chen, M.; Clark, T.; Anilkumar, K.; Huang, F. Fitness costs and stability of Cry1Ab resistance in sugarcane borer, *Diatraea saccharalis* (F.). *J. Invertebr. Pathol.* **2014**, *117*, 26–32. http://dx.doi.org/10.1016/j.jip.2014.01.007

Zhang, W.; Ma, L.; Zhong, F.; Wang, Y.; Guo, Y.; Lua, Y.; Liang, G. Fitness costs of reproductive capacity and ovarian development in a Bt-resistant strain of the cotton bollworm *Helicoverpa armigera* (Hübner) (Lepidoptera: Noctuidae). *Pest Manag. Sci.* **2015**, *71*, 870–877.

http://doi.org/10.1002/ps.3900

Zhang, W.N.; Ma, L.; Wang, B.J.; Chen, L.; Khaing, M.M.; Lu, Y.H.; Liang, G.M.; Guo, Y.Y. Reproductive cost associated with juvenile hormone in Bt-resistant strains of *Helicoverpa armigera* (Lepidoptera: Noctuidae). *J. Econ. Entomol.* **2016**, *109*, 2534–2542. http://doi.org/10.1093/jee/tow233

Zhao, X.C.; Wu, K.M.; Liang, G.M.; Guo, Y.Y. Modified female calling behaviour in Cry1Ac-resistant *Helicoverpa armigera* (Lepidoptera: Noctuidae). *Pest. Manag. Sci.* **2009**, *65*, 353–357. http://doi.org/10.1002/ps.1697
